# Effect of ambulatory versus hospital treatment for gestational diabetes or hyperglycemia on infant mortality rates: a systematic review

**DOI:** 10.1590/1516-3180.2013.1315560

**Published:** 2013-10-01

**Authors:** Marilza Vieira Cunha Rudge, Silvana Andréa Molina Lima, Regina Paolucci El Dib, Gabriela Marini, Claudia Magalhães, Iracema de Mattos Paranhos Calderon

**Affiliations:** I MD, PhD. Full Professor, Department of Gynecology and Obstetrics, Faculdade de Medicina de Botucatu (FMB), Universidade Estadual Paulista (Unesp), Botucatu, São Paulo, Brazil.; II PhD. Professor, Department of Nursing, Faculdade de Medicina de Botucatu (FMB), Universidade Estadual Paulista (Unesp), Botucatu, São Paulo, Brazil.; III PhD. Professor, Department of Anaesthesiology, Faculdade de Medicina de Botucatu (FMB), Universidade Estadual Paulista (Unesp), Botucatu, São Paulo, Brazil.; Research Collaborator of the McMaster Institute of Urology, at St. Joseph's Healthcare, Hamilton, Canada.; IV Postgraduate Student, Department of Gynecology and Obstetrics, Faculdade de Medicina de Botucatu (FMB), Universidade Estadual Paulista (Unesp), Botucatu, São Paulo, Brazil.; V MD, PhD. Professor, Department of Gynecology and Obstetrics, Faculdade de Medicina de Botucatu (FMB), Universidade Estadual Paulista (Unesp), Botucatu, São Paulo, Brazil.

**Keywords:** Diabetes, gestational, Ambulatory care, Hospitalization, Review [publication type], Clinical trials as topic, Diabetes gestacional, Assistência ambulatorial, Hospitalização, Revisão, Ensaios clínicos como assunto

## Abstract

**CONTEXT AND OBJECTIVE:**

Pregnancies complicated by diabetes are associated with increased neonatal and maternal complications. The most serious maternal complication is the risk of developing type 2 diabetes, 10-12 years after the delivery. For rigorous control over blood glucose, pregnant women are treated through ambulatory management or hospitalization. The aim of this study was to evaluate the effectiveness of ambulatory management versus hospitalization in pregnancies complicated by diabetes or hyperglycemia.

**DESIGN AND SETTING:**

Systematic review conducted in a public university hospital.

**METHODS:**

A systematic review of the literature was performed and the main electronic databases were searched. The date of the most recent search was September 4, 2011. Two authors independently selected relevant clinical trials, assessed their methodological quality and extracted data.

**RESULTS:**

Only three studies were selected, with small sample sizes. There was no statistically significance different between ambulatory management and hospitalization, regarding mortality in any of the subcategories analyzed: perinatal and neonatal deaths (relative risk [RR] 0.65; 95% confidential interval [CI]: 0.11 to 3.84; P = 0.63); neonatal deaths (RR 0.29; 95% CI: 0.01 to 6.07; P = 0.43); and infant deaths (RR 0.29; 95% CI: 0.01 to 6.07; P = 0.43).

**CONCLUSIONS:**

This review, based on studies with high or moderate risk of bias, showed that there was no statistically significant difference between ambulatory management and hospital care, regarding reduction of mortality rates in pregnancies complicated by diabetes or hyperglycemia. It also suggested that there is a need for further randomized controlled trials on this issue.

## Abstract (pt)

**CONTEXTO E OBJETIVO:**

Gestações complicadas pelo diabetes estão associadas com aumento das complicações neonatais e maternas. A complicação mais grave materna é o risco de desenvolver diabetes tipo 2 após 10-12 anos do parto. Para o controle rigoroso da glicose no sangue, as mulheres grávidas são tratadas de forma ambulatorial ou com internações hospitalares. O objetivo deste estudo é avaliar a efetividade do tratamento ambulatorial versus hospitalização em gestações complicadas por diabetes ou hiperglicemia.

**TIPO DE ESTUDO E LOCAL:**

Revisão sistemática conduzida em hospital universitário público.

**MÉTODOS:**

Uma revisão sistemática da literatura foi realizada e as principais bases de dados eletrônicas foram pesquisadas. A data da pesquisa mais recente foi 4 de setembro de 2011. Dois autores selecionaram independentemente os ensaios clínicos relevantes, avaliaram a qualidade metodológica e extraíram os dados.

**RESULTADOS:**

Apenas três estudos foram selecionados, com tamanho de amostra pequeno. Não houve diferença estatisticamente significativa entre o tratamento ambulatorial versus hospitalização em relação à mortalidade em nenhuma das subcategorias analisadas: mortes perinatais e neonatais, (risco relativo [RR] 0,65; 95% de intervalo de confiança [IC] 0,11-3,84, P = 0,63); morte neonatal (RR 0,29, IC 95% 0,01-6,07, P = 0,43), e óbitos infantis (RR 0,29, IC 95% 0,01-6,07, P = 0,43).

**CONCLUSÕES:**

Com base em estudos com risco de viés alto ou moderado, esta revisão demonstrou que não há diferença estatisticamente significante entre o tratamento ambulatorial comparado com o hospitalar na redução das taxas de mortalidade em gestações complicadas por diabetes ou hiperglicemia. Esta revisão sistemática também sugere a necessidade de mais ensaios clínicos randomizados sobre o assunto.

## INTRODUCTION

Gestational diabetes mellitus (GDM) is defined as any degree of glucose intolerance with onset or first recognition during pregnancy. Although most cases resolve with delivery, this definition is still applied regardless of whether the condition persists after pregnancy, and it excludes the possibility that unrecognized glucose intolerance may have antedated or begun concomitantly with the pregnancy. According to the American Diabetes Association (ADA),^1^ this definition has facilitated a uniform strategy for detection and classification of GDM, but its limitations have been recognized for several years.^1^

The Hyperglycemia and Adverse Pregnancy Outcomes (HAPO) study demonstrated that the risk of adverse maternal, fetal and neonatal outcomes continuously increased as a function of maternal glycemia at 24-28 weeks, even within ranges that were previously considered to be normal for pregnancy.^2^ Rudge et al^3^ investigated the oral glucose tolerance test (OGTT; 100 g) and the glucose profile (GP) in association with diagnoses of GDM, and unexpectedly identified four groups of pregnant women: IA = normal OGTT + normal GP; IB = normal OGTT + abnormal GP; IIA = abnormal OGTT + normal GP; and IIB = abnormal OGTT + abnormal GP. According to the ADA's criteria, groups IIA and IIB are patients with diabetes, while individuals in group IB are not because their OGTT is normal. The risks of macrosomia in groups IB, IIA and IIB were statistically similar, and the perinatal mortality rate was 10 times higher than in group IA (not diabetes patients). Thus, these authors concluded that the adverse outcomes observed were due to hyperglycemia, which was present in group IB but underdiagnosed by means of OGTT alone. Since then, normal OGTT and abnormal GP have become the criteria used to diagnose mild hyperglycemia (group IB) and to determine the most appropriate treatment for these pregnant women.^4^

Despite the recognition that hyperglycemia in pregnancy is associated with both adverse maternal and adverse neonatal outcomes, there has been uncertainty about the benefits of treating these patients. The Australian Carbohydrate Intolerance Study in Pregnant Women (ACHOIS) concluded that controlling maternal hyperglycemia reduced serious perinatal morbidity and might also improve women's health-related quality of life.^5^ The Maternal and Fetal Medicine Units Network (MFMUN) undertook a multicenter randomized trial comparing diet and insulin therapy versus no specific treatment, among women with mild hyperglycemia in pregnancy.^6^ Regardless of the diagnostic test, it is clear that maternal hyperglycemia needs to be controlled. 

It is well established that the principal factor responsible for success in a diabetic pregnancy is strict glycemic control, with particular emphasis on maintaining blood glucose level within the normal range.^7^ To obtain such control, these women used to be subjected to frequent and prolonged hospitalizations.^8^ In the 1980s, a new therapeutic scheme for controlling maternal diabetes was proposed;^9-11^ it entailed intensive care monitoring of high-risk perinatal patient with diabetes. According to one report, ambulatory management of pregnant patients with diabetes led to a significant rise in perinatal mortality among pregestational women with diabetes.^7^ Conversely, other reports demonstrated a lower mortality rate but a high rate of maternal and neonatal morbidity.^12^

In Brazil, a study conducted among pregnant women with diabetes showed that maternal and fetal outcomes such as maternal and perinatal mortality did not differ significantly between ambulatory management and hospitalization.^13^ However, the total costs of prenatal, delivery and postpartum care were significantly higher in the diabetic pregnant hospitalization group than in the ambulatory management group.^14^

To the best of our knowledge, no systematic review comparing ambulatory management versus hospitalization in GDM or hyperglycemia cases has been conducted. Thus, it is important to investigate the best treatment strategy (ambulatory or hospital care) for pregnant women with diabetes. In this paper, we summarize the existing knowledge using standardized criteria, in order to perform a systematic review of the interventions.^15,16^

### OBJECTIVE

To evaluate the effectiveness of ambulatory management versus hospitalization in GDM or hyperglycemia cases. 

### METHODS

This systematic review of the literature on intervention studies was conducted in accordance with the PRISMA (Preferred Reposting Items for Systematic Reviews and Meta-analysis) statement.^17^

*Eligibility criteria*

We took into consideration all randomized and quasi-randomized controlled clinical trials evaluating ambulatory management versus hospitalization in pregnancies complicated by diabetes or hyperglycemia. 

The outcomes assessed were perinatal, neonatal and infant mortality. Studies were excluded from the review if they were duplicate publications on a study that had already been included, animal studies, case reports or review articles. 

*Search*

**Chart 1:**
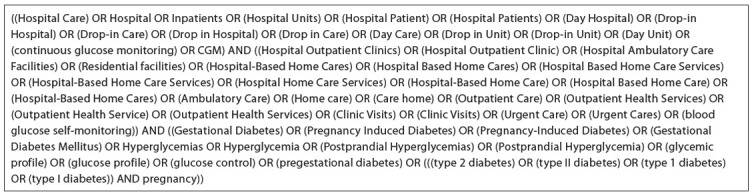
Search strategy for all electronic databases

There was no restriction on language, year of publication or publication status. The search was performed in the following electronic databases: the Cochrane database of clinical trials (CENTRAL, 2013, issue 1), PubMed (1966-2013), Embase (1980-2013), Lilacs (1982-2013) and Scientific Electronic Library Online (SciELO). The databases were searched for available published and unpublished studies up to January 27, 2013. The search was conducted using multiple combinations of the following key words: hospital care, inpatients, day hospital, drop-in hospital, hospital outpatient clinics, hospital outpatient clinic, hospital ambulatory care facilities, hospital-based home care, outpatient care, gestational diabetes, pregnancy-induced diabetes and gestational diabetes mellitus (Chart 1). 

In addition, a manual search of the bibliographic pages of the selected articles and the content pages of major diabetes journals was conducted. Study authors were contacted to identify additional studies. 

*Study selection and data extraction*

The titles and abstracts were reviewed by two researchers (MVCR and RED) to identify potentially relevant papers. The papers were obtained and independently read in full by the two reviewers. Differences were resolved by discussion and a third party (IMPC), if necessary. Reasons for exclusion were identified. The data were also extracted independently by MVCR and RED based on the inclusion and exclusion criteria defined above.

*Risk of bias in individual studies*

A risk of bias table, which is a Cochrane measurement tool used to assess the methodological quality of clinical trials, was used as a guide to conduct this systematic literature review.^15,16^ We used the following six separate criteria: random sequence generation; allocation concealment; blinding; incomplete outcome data; selective reporting; and other sources of bias. 

*Summary measurements and synthesis of results*

For dichotomous data, we used relative risk (RR) as the effect measurement, with 95% confidence intervals (CI), along with a fixed-effects model. The null hypothesis of homogeneity across individual studies was tested using the chi-square test and the I^2^ value. 

### RESULTS

The electronic search yielded a total of 549 published papers from the electronic databases, and this total was screened using their titles and abstracts. Six full-text articles were retrieved for further consideration. Cross-checking of the references and manual searches did not yield any additional studies for inclusion. Of these six full-text articles, three studies met the inclusion criteria.^18-20^ However, Brooten et al.^18^ reported data including not only GDM and pre-gestational diabetes but also chronic hypertension and pregnancies at risk of preterm birth. Thus, we contacted the main author of this study^18^ and requested the data separately, so as to include only the data for GDM and pre-gestational diabetes in the analysis. Unfortunately, we did not receive any response. 

**Figure 1:**
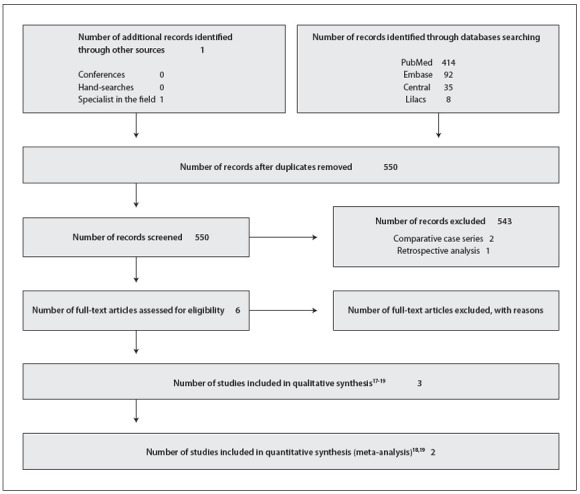
Flowchart of the systematic review.

Three studies were excluded from this review: two studies^12-21^ were classified as comparative case series; and one study^22^ was presented as a retrospective analysis (Figure 1). Only three studies were selected. The studies included presented data on a total of 736 pregnant women with diabetes. The study by Nachum et al.^20^ evaluated the largest number of participants (92.5%), followed by Brooten et al.^18^ with 5.7% and Stubbs et al.^19^ with 1.7%. Brooten et al.^18^ followed the patients for 12 months after delivery using the multiple affect adjective form, and the patients were evaluated by a physician six weeks after delivery. Stubbs et al.^19^ included a follow-up at least ten days after routine hospital admission. Although Nachum et al.^20^ reported on an eight-year prospective controlled study, the exact follow-up period for each outcome after delivery was unclear.

The participants in the study by Brooten et al.^18^ were randomized into two groups. The first was the home care group, in which the women received standard prenatal and postpartum care for high-risk pregnancies from the hospital clinic, from resident and staff physicians. However, half of the prenatal clinical care provided by physicians was replaced with care delivered in the woman's home by specialist nurses with master's degrees. The control and intervention groups were scheduled to receive the same total number of prenatal visits. During the postpartum period, the women in the intervention group were contacted by telephone weekly for eight weeks by a team of nurses to monitor their physical state. The second group was the hospital care group, in which standard prenatal and post partum care was given to high-risk patients at the hospital clinic, by residents and staff physicians. Women with pre-gestational diabetes mellitus were seen weekly or every other week if their diabetes mellitus was less severe. This was continued until 33 weeks of gestation and then twice a week until delivery. Pregnant women with diabetes were seen every other week until 35 weeks of gestation and then weekly until delivery. All the women were educated by the nursing staff regarding their pregnancies and the associated risks.

Stubbs et al.^19^ allocated patients to programs of either selfmonitored blood glucose treatment (home care group) or conventional treatment (hospital care group) in weeks 30-31. Seven patients were placed in the home care group, and they measured their blood glucose at home with "Dextrostix" (Ames) using an "Eyetone" meter. The six patients in the hospital care group were checked for urinary glucose four times a day, and their blood glucose levels were measured at clinic visits every two weeks.

Nachum et al.^20^ did not randomize the singleton women with diabetes, for whom therapy was started before they reached 34 gestational weeks. The patients were routinely managed by means of repeated hospitalizations between 1986 and 1989, and ambulatory care was provided between 1990 and 1993.

Brooten et al.^18^ evaluated maternal outcomes using the Multiple Affect Adjective checklist, which included items such as anxiety, depression and hostility. The patients' satisfaction with their care was measured using the La Monica-Oberst Patient Satisfaction Scale. The authors also assessed hospitalization during pregnancy and the length of stay for delivery, as well as acute care visits and hospital stays after delivery (readmissions). For the infant outcomes, the authors evaluated mortality, prematurity, birth weight and hospitalization. Stubbs et al.^19^ assessed blood glucose concentration of metabolite, infant birth weight and mortality. Nachum et al.^20^ measured glycemic control, birth data, perinatal and neonatal mortality and major congenital abnormalities. 

### Risk of bias in the studies included

**Figure 2:**
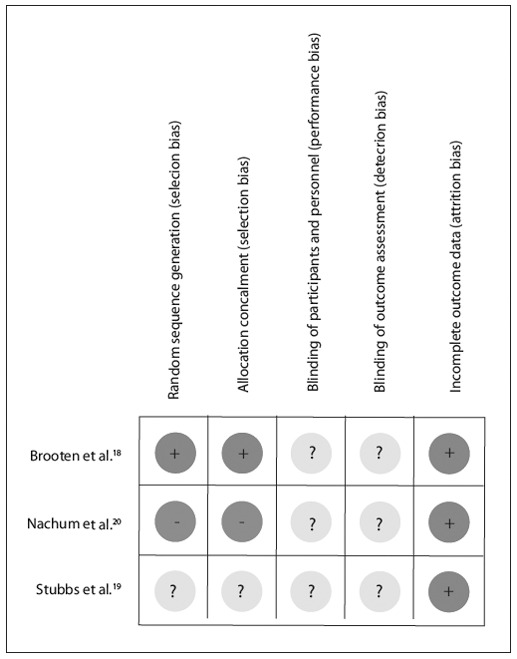
Risk of bias summary: review of authors' judgments about each risk of bias item for each study included.

None of the measurements in Brooten et al.^18^ Stubbs et al.^19^ and Nachum et al.^20^ were clear regarding examiner blinding to treatment groups. With regard to allocation generation, the study by Brooten et al.^18^ presented a low risk of bias, given that the allocation sequence was prepared in advance by a statistician using a list of random numbers. Conversely, the study by Stubbs et al.^19^ had a moderate risk of bias because no method was reported. The study by Brooten et al.^18^ reported that a sealed envelope sequence was used, thus giving a low risk of bias. The study by Stubbs et al.^19^ was unclear with regard to randomization because there was no description of this in their paper. Nachum et al.^20^ did not randomize their patients and they justified this in terms of their population's cultural background. There were no incomplete outcome reports in the studies included.^18-20^ Overall, all the studies included presented a moderate risk of bias, with the exception of Nachum's 2001 study, which was ranked as presenting a high risk of bias (Figure 2).

### Effectiveness of interventions

*Meta-analysis*

**Figure 3:**
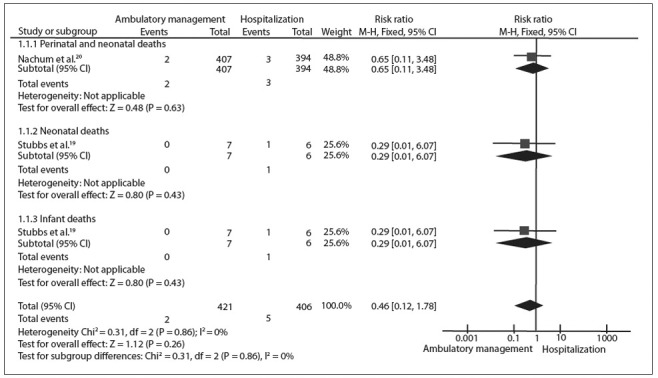
Meta-analysis comparing ambulatory management versus hospitalization with regard to mortality.

There was no statistically significant difference between ambulatory management and hospitalization with regard to mortality in any of the subcategories analyzed: perinatal and neonatal deaths (RR 0.65; 95% CI: 0.11 to 3.84; P = 0.63);^20^ neonatal deaths (RR 0.29; 95% CI: 0.01 to 6.07; P = 0.43);^19^ and infant deaths (RR 0.29; 95% CI: 0.01 to 6.07; P = 0.43).^19^ There was also no statistically significant difference between ambulatory management and hospitalization with regard to the overall effect of deaths (RR 0.46; 95% CI: 0.12 to 1.78; P = 0.26),^20,21^ as indicated by the diamonds in Figure 3. 

### DISCUSSION

This systematic review provides up-to-date but limited evidence supported by three studies,^18-20^ regarding the effects of ambulatory management versus hospitalization among pregnant women with GDM or hyperglycemia. The studies showed clinical and methodological differences. Emails were sent to the authors of the studies included, with the aim of clarifying some methodological issues that remained unclear; however, no authors had contacted us by the time of this paper's completion. 

Diabetes mellitus has reached epidemic proportions worldwide, and its incidence continues to increase. Considering the increasing number of patients diagnosed with diabetes and associated complications, the complexity of care for this population can be particularly challenging. The prevalence of diabetes mellitus is increasing worldwide, but especially in developing countries. Improvements in maternal and perinatal outcomes among pregnant women with diabetes have been widely documented over the last two decades (American Diabetes Association).^22^ The current evidence relating to mortality in ambulatory versus hospitalization management for pregnant women with diabetes was studied in this systematic review. However, despite an exponential increase of published papers and studies on patients with diabetes over the last decade, many doubts still exist in relation to what constitutes the best form of care for women with diabetes in the settings of home and hospitalbased therapy. 

One of the limitations of this review was the small number of clinical trials included and the low event rate observed in the studies included. None of the studies included reported how the sample size was calculated or whether the sample had significant statistical power. Furthermore, the duration of follow-up in the studies included was too short and perhaps not a true representation of total infant mortality. A reasonable follow-up period for this outcome would be at least one year; however, whether any of the studies followed up the children for such a long time is questionable. Therefore, neonatal mortality rates would be more appropriate. 

Ambulatory management ought to be the first choice for treating women with diabetes during pregnancy because it provides a combination of effective and inexpensive procedures, in comparison with hospitalization. However, the results from this review confirmed that there is a need for further randomized clinical trials (RCTs) to ensure greater understanding of both precision and care during the prenatal period. 

The Diabetes Research Centre of the Perinatal Hospital of Botucatu Medical School, Universidade Estadual Paulista (Unesp), Brazil, is already conducting an RCT to analyze the effects of ambulatory management versus hospitalization on the maternal and perinatal outcomes of pregnancies complicated by diabetes or hyperglycemia and the cost-effectiveness of the care provided for these women.

### CONCLUSION

This review, based on studies with high or moderate risk of bias, showed that there was no statistically significant difference between ambulatory management and hospital care with regard to reduction of mortality rates among GDM cases. This systematic review also suggested that there is a need for future randomized controlled trials comparing ambulatory management versus hospital care for pregnant women with diabetes. It would be extremely helpful if outcome measurements such as hospitalization during pregnancy, length of stay for delivery, acute care visits and hospital stays after delivery due to maternal outcomes were to be standardized. Additionally, in terms of infant outcomes, measurements such as mortality, prematurity, birth weight and hospitalization should be gathered. It would also be helpful if outcome data were gathered for subgroup analysis, including on mortality and comorbidities.
